# Co‐infection of Hepatitis A and E With Atypical Manifestation: A Case Report

**DOI:** 10.1002/ccr3.72857

**Published:** 2026-06-02

**Authors:** Umaimah Batool Mirza, Habib Ul Rahman Kalhoro, FNU Umna, Ahmed Asad Raza, Abedin Samadi

**Affiliations:** ^1^ Jinnah Sindh Medical University Karachi Pakistan; ^2^ Kabul University of Medical Sciences Kabul Afghanistan

**Keywords:** acute viral hepatitis, co‐infection, hepatitis A, hepatitis E, pediatric hepatitis, starry sky liver

## Abstract

Hepatitis A virus (HAV) and hepatitis E virus (HEV) are common causes of acute viral hepatitis in developing countries where inadequate sanitation and contaminated water supplies facilitate fecal–oral transmission. Although both infections are usually self‐limiting, co‐infection may result in atypical presentations and pose diagnostic challenges. We report the case of a previously healthy 14‐year‐old girl who presented with a 5‐day history of nausea, vomiting, and anorexia, followed by sudden‐onset jaundice and dark urine. She denied fever, abdominal pain, pruritus, bleeding manifestations, or altered mental status. Physical examination revealed marked icterus without hepatosplenomegaly or stigmata of chronic liver disease. Laboratory investigations demonstrated significant hyperbilirubinemia and elevated liver transaminases. Serological testing confirmed acute HAV and HEV co‐infection with positive anti‐HAV IgM and anti‐HEV IgM antibodies. Abdominal ultrasonography showed a normal‐sized liver with altered echogenicity and a characteristic “starry sky” appearance, along with gallbladder wall thickening without calculi or sludge. The patient received supportive treatment, including intravenous fluids, antiemetics, and symptomatic care. Her clinical condition improved steadily, with resolution of symptoms and progressive normalization of liver function tests during hospitalization. She was discharged in stable condition and remained asymptomatic at 14‐day follow‐up, with complete biochemical and radiological recovery. This case highlights that HAV–HEV co‐infection can present with isolated jaundice and minimal systemic symptoms despite significant hepatic involvement. Recognition of characteristic imaging findings and timely serological evaluation can facilitate early diagnosis. Favorable outcomes can be achieved with supportive management, while prevention through vaccination, safe drinking water, and improved hygiene remains essential in endemic regions.

## Key Clinical Message

Co‐infection with hepatitis A and E may present subtly in healthy adolescents, showing isolated jaundice and a distinctive “starry‐sky” hepatic ultrasound pattern. Early recognition enables timely diagnosis and supportive therapy ensures full recovery, emphasizing prevention through vaccination and hygiene.

**Abbreviations**
HAV: hepatitis A virus
HBV: hepatitis B Virus
HEV: hepatitis E virus
INJ: injection
LFT: liver function test
OD: Omni Die (Latin for “once daily”)
PCR: polymerase chain reaction
RNA: ribonucleic acid
TDS: Ter Die Sumendum (Latin for “to be taken three times a day”)

## 1 Introduction

Developing nations such as Pakistan face a significant risk of infection from fecal‐oral pathogens like the hepatitis A virus (HAV) and hepatitis E virus (HEV), attributable to poor sanitary conditions and a lack of hygienic practices [1]. Hepatitis A is a well‐known cause of hepatitis around the world. It usually causes a short‐term, self‐limiting disease for about 4–7 weeks. The presentation is asymptomatic among young children, but acute liver failure and fulminant hepatitis in the elderly are among the symptoms, which also include fever, malaise, lack of appetite, diarrhea, nausea, stomach discomfort, dark urine, and jaundice [2]. Hepatitis E virus also develops a self‐limiting infection, but if it infects immunocompromised, or patients with pre‐existing liver disease. It can progress to chronic hepatitis, cirrhosis, or acute liver failure [3]. HEV infection develops severe complications of liver failure and death if it infects pregnant women, especially in the third trimester [4].

A retrospective study at Jinnah Postgraduate Medical Center, Karachi, Pakistan, reviewed the cases of hepatitis from 1987 to 2007. Hepatitis E and hepatitis A have been reported to be (20.2%) and (3.5%) prevalent, respectively [5]. According to the World Health Organization, the mortality rates of HAV and HEV are found to be (0.5%) and (3.3%), respectively [6]. Co‐infection of HAV and HEV is a relatively rare but clinically significant phenomenon in Pakistan, where viral hepatitis is endemic. This case highlights an unusual presentation of HAV and HEV co‐infection in another healthy 14‐year‐old girl, who demonstrated a subtle but progressive clinical course culminating in severe jaundice and deranged LFTs.

## 2 Case History

A 14‐year‐old South Asian school‐going girl, previously in good health with no known medical conditions, presented to the emergency department accompanied by her mother with a primary complaint of progressively deepening jaundice over the past 2 days, preceded by a 5‐day history of nausea, repeated episodes of non‐bilious vomiting, and marked loss of appetite. She reported that the illness began insidiously, initially as mild queasiness and poor oral intake, which she attributed to eating street food after school. She continued attending school during the first 3 days of illness but noted worsening fatigue. On the fourth day, she noticed her eyes turning yellow and her urine becoming dark in color; this prompted her family to seek medical attention. She denied fever, pruritus, abdominal pain, distension, altered bowel habits, joint pain, bleeding manifestations, or changes in mental status. There was no history of similar symptoms in family members, no recent travel, and no use of herbal or over‐the‐counter medications. Her immunizations were up to date, and there was no known exposure to patients with jaundice.

On examination, she was alert, oriented, and afebrile, with stable vital signs. She had deep icterus but no pallor, cyanosis, lymphadenopathy, skin rashes, or signs of bleeding. Abdominal examination revealed no hepatosplenomegaly, tenderness, or distension, and there was no evidence of ascites. Cardiovascular, respiratory, and neurological examinations were unremarkable. There were no stigmata of chronic liver disease such as spider angiomas, palmar erythema, or gynecomastia.

Initial laboratory evaluation revealed a normocytic normochromic blood picture, a normal total leukocyte count, and mild thrombocytopenia. Liver function tests were significantly deranged, showing elevated total bilirubin with raised direct and indirect fractions, and markedly elevated transaminases, as detailed in Table 1. Serum electrolytes, renal function tests, coagulation profile, and C‐reactive protein were within normal limits. Acute viral hepatitis serology demonstrated positive anti‐HAV IgM and anti‐HEV IgM, confirming acute co‐infection.

**TABLE 1:** Liver function test (LFT) results of the patient on admission and after 2 days of hospitalization.

Test description	Result on admission	Result after 2 days of admission	Results after 14 days of admission	Reference value
Serum bilirubin (direct)	3.6 mg/dL	4.0 mg/dL	0.47 mg/dL	≤ 0.2 mg/dL
Serum bilirubin (indirect)	1.4 mg/dL	1.5 mg/dL	0.25 mg/dL	≤ 1.1 mg/dL
ALT	2851 IU/L	1921 IU/L	478 IU/L	< 33 IU/L
AST	2698 IU/L	1740 IU/L	256 IU/L	< 32 IU/L
GGT	62 U/L	42 U/L	29 U/L	5–36 U/L
ALP	260 U/L	234 U/L	178 U/L	57–254 U/L
Serum albumin	4.4 g/dL	3.6 g/dL	3.1 g/dL	2.5–3.4 g/dL
Serum globulin	3.2 g/dL	2.6 g/dL	2.3 g/dL	1.5–2.5 g/dL

*Note:* This table presents the biochemical parameters related to liver function observed in the patient at the time of hospital admission, after 48 h and after 14 days. Significant elevations in alanine aminotransferase (ALT) and aspartate aminotransferase (AST) levels indicate acute hepatocellular injury. Serum bilirubin (direct and indirect) levels also increased, correlating with the clinical finding of jaundice. Gamma‐glutamyl transferase (GGT) and alkaline phosphatase (ALP) levels were mildly elevated, while serum albumin and globulin levels were slightly above the reference range initially. The trends over these days reflect a gradual biochemical improvement under conservative management. Reference values are provided for comparison.

**FIGURE 1:**
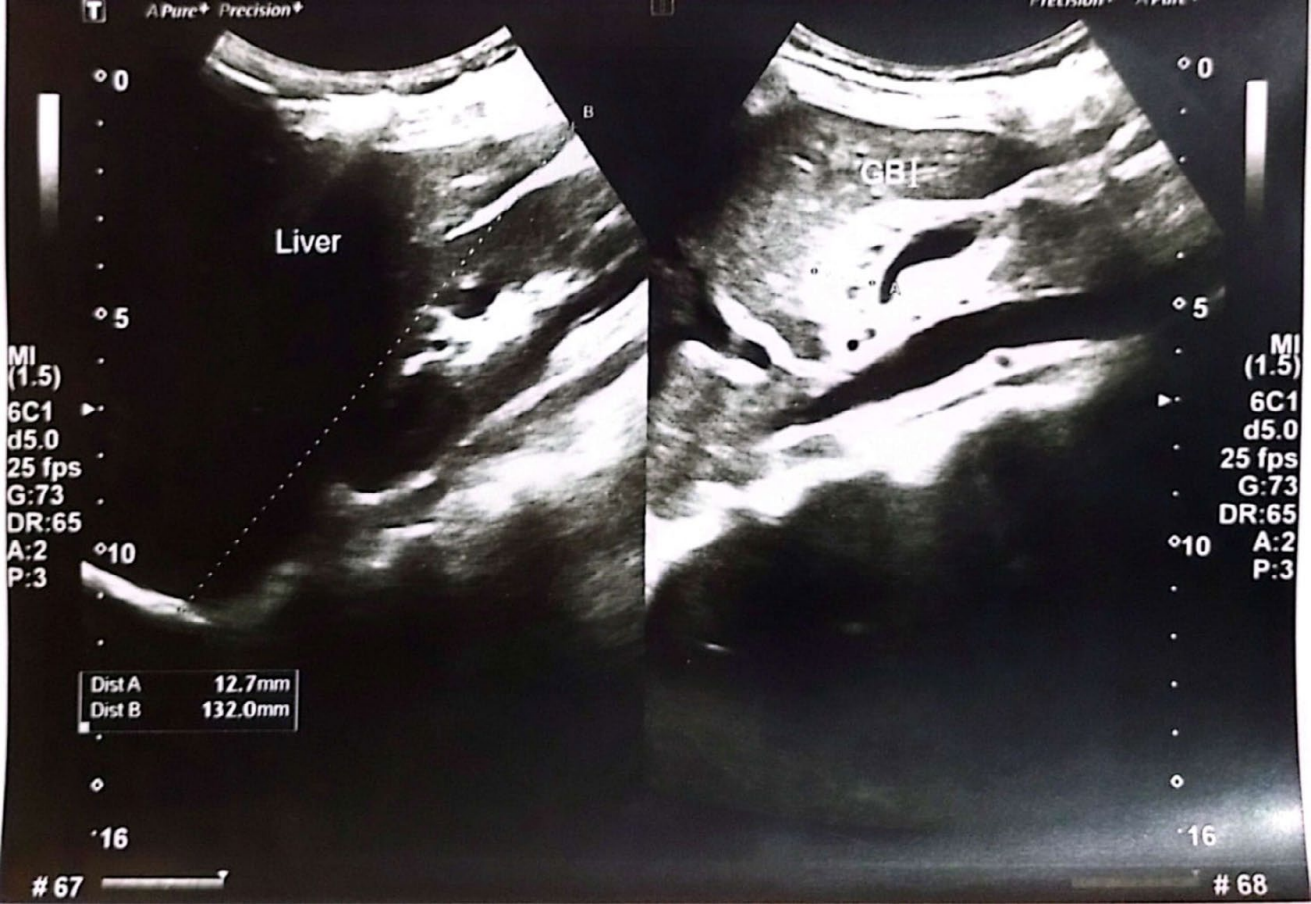
Abdominal ultrasound showing a normal‐sized liver with altered echogenicity and characteristic “starry sky” appearance. The hyperechoic portal triads with periportal edema and reduced background hepatic echogenicity are indicative of acute hepatitis changes.

**FIGURE 2:**
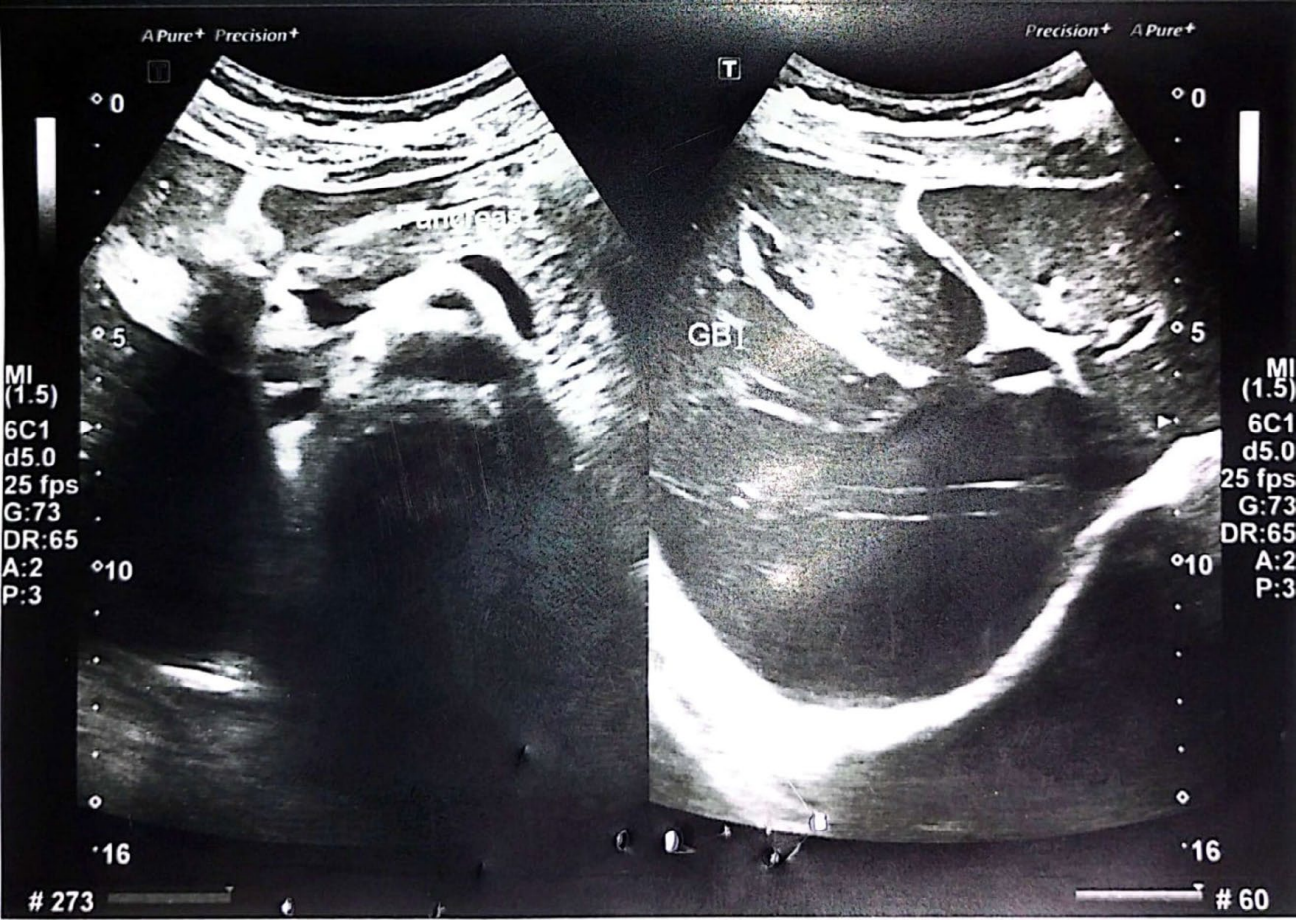
Close‐up ultrasound view of the periportal regions demonstrating the starry sky pattern. Bright portal vein walls contrast with the darkened hepatic parenchyma due to inflammation and fluid accumulation.

**FIGURE 3:**
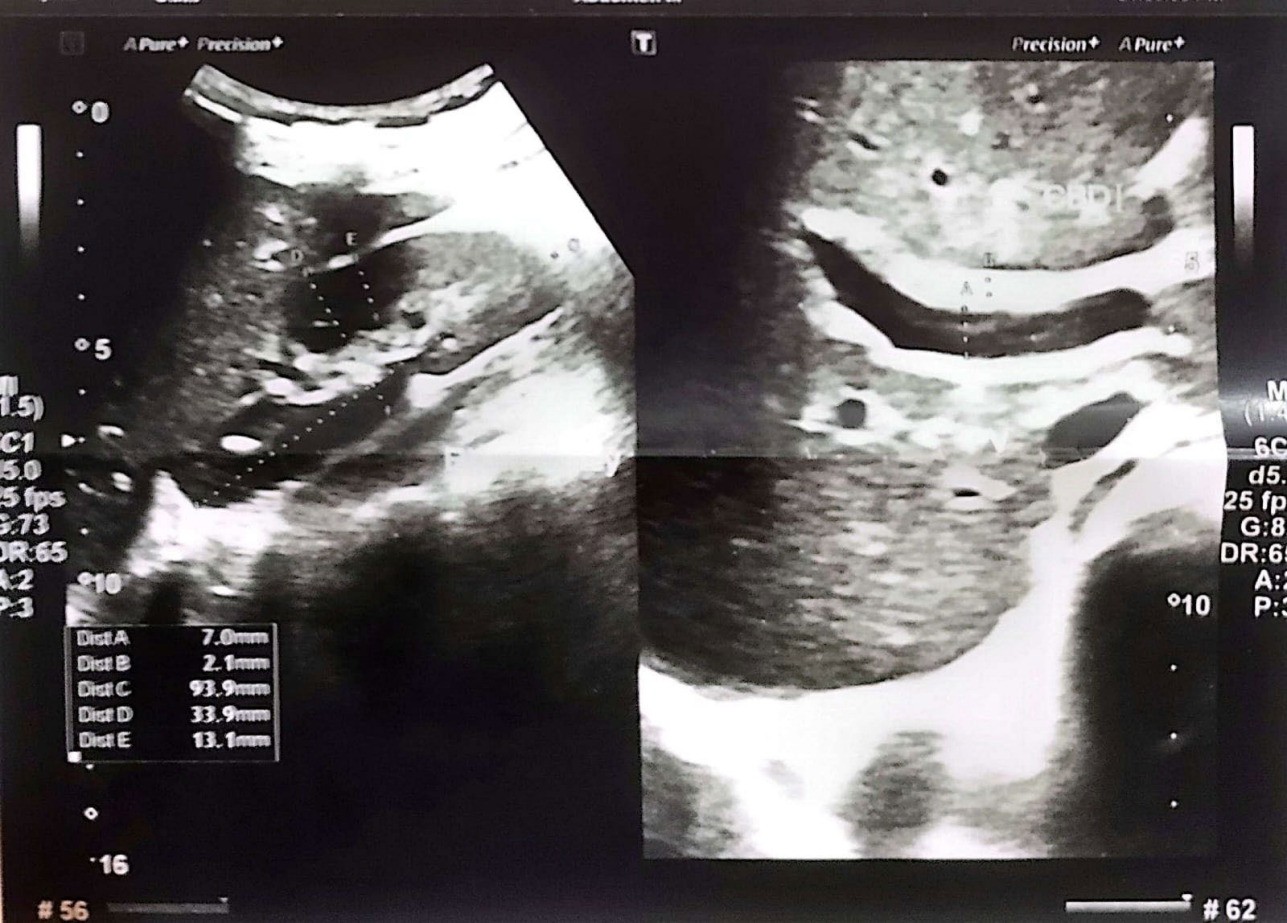
Ultrasound image showing gallbladder wall thickening without calculi or sludge. The wall measures 12.7 mm in thickness, consistent with acute cholecystitis secondary to viral hepatitis.

Abdominal ultrasonography revealed a normal‐sized liver (13.2 cm) with altered echogenicity and a characteristic “starry sky” pattern, consistent with acute hepatitis changes. The gallbladder wall was thickened to 12.7 mm without evidence of calculi or sludge. No ascites or focal hepatic lesions were noted. These findings are illustrated in Figures 1, 2, 3.

She was admitted for supportive management. Treatment included intravenous dextrose saline (80 mL/h), ondansetron 8 mg IV TDS for nausea, paracetamol 1 g IV TDS for symptomatic relief, and omeprazole 40 mg IV OD. Her oral intake was monitored closely, and she was gradually transitioned to oral medications—ondansetron 8 mg TDS and omeprazole 40 mg OD—which were prescribed for 15 days on discharge. By the third day of hospitalization, her nausea had subsided, her appetite returned, and the intensity of her jaundice began to diminish. Liver function tests showed progressive improvement.

She was discharged on Day 9 of hospitalization in stable condition, with advice on maintaining strict personal and food hygiene, avoiding street food, and consuming safe drinking water. At the scheduled 14‐day follow‐up, she reported complete resolution of symptoms, had resumed school and normal activities, and her repeat liver function tests and abdominal ultrasound were entirely normal. The chronological summary of her illness, interventions, and recovery is presented in Table 2.

**TABLE 2:** Chronological timeline of clinical presentation, investigations, interventions, and outcomes.

Date/illness day	Events and symptoms	Clinical findings/tests	Interventions	Outcome
Day 1	Nausea, vomiting, loss of appetite begin	No jaundice	None	Symptoms persist
Day 4	Yellowing of eyes and dark urine appear	Deep jaundice, no fever or abdominal pain	Presented to ER, admitted	Hospitalization initiated
Day 5 (admission)	Severe jaundice, fatigue	Elevated bilirubin & transaminases, HAV & HEV IgM positive, ultrasound: “starry sky” liver	IV fluids, ondansetron, paracetamol, omeprazole	Monitoring started
Day 8	Symptoms improving	Stable vitals, declining LFTs	Continued supportive care	Planned discharge
Day 9 (discharge)	Minimal symptoms	Clinically stable	Switched to oral meds for 15 days	Outpatient follow‐up advised
Day 14 follow‐up	No symptoms	LFTs normal, ultrasound normal	None	Full recovery

### 2.1 Differential Diagnosis

The differential diagnoses for this presentation included acute viral hepatitis (A–E), drug‐induced liver injury, autoimmune hepatitis, Wilson's disease, hemolytic disorders, and obstructive or cholestatic hepatobiliary disease such as gallstone‐related hepatitis or cholangitis.

### 2.2 Patient Perspective

“At the start, I thought it was just an upset stomach from something I ate, so I didn't tell my mother right away. When my eyes started turning yellow, I felt frightened, and I was worried it might be something serious. Staying in the hospital was scary at first, but the doctors explained everything to me and my family, which made me feel safe. I started feeling better after a few days, and it was a relief when they told me my blood tests and scans were back to normal. Now I'm more careful with what I eat and drink, and I always make sure my water is clean.”

## 3 Conclusion

This case underscores the need for clinicians to consider HAV and HEV co‐infection in patients who exhibit acute hepatitis, especially in endemic regions and developing countries like Pakistan. The subtle presentation of jaundice and the characteristic “starry sky” ultrasound pattern are important diagnostic clues. Improved sanitation, public health measures, and vaccination against HAV remain crucial strategies in preventing such infections.

## 4 Limitations

This case report has several limitations. Firstly, as a single case study, it may not be generalizable to the broader population due to individual variability in clinical presentation and disease progression. Secondly, the absence of serial imaging or liver biopsy limits a more detailed assessment of the hepatic changes during recovery. Thirdly, diagnosis relied solely on serology (anti‐HAV IgM and anti‐HEV IgM); molecular confirmation via HAV and HEV RNA PCR was not performed. PCR testing would have strengthened diagnostic specificity, quantified viral load, and excluded potential IgM cross‐reactivity. This represents a meaningful methodological limitation that should be addressed in future investigations of atypical hepatitis presentations.

## 5 Discussion

HAV and HEV co‐infection is associated with more severe clinical outcomes than either infection alone. A study from southern Mexico reported a co‐infection prevalence of 10% among children with acute hepatitis [7]. A hospital‐based study from Uttarakhand, India, found a prevalence of 5.9% for dual HAV/HEV infection among acute viral hepatitis cases [8]. A study from Bangladesh reported a co‐infection rate of 5.3% in a pediatric population [9]. Additionally, during and after monsoon seasons, seropositive results for both HAV and HBV were identified in 5.3% of dengue virus patients, highlighting overlapping water‐borne transmission dynamics in endemic settings [10].

Co‐infection of HAV and HEV is facilitated by shared transmission pathways and overlapping immunopathogenic mechanisms. Both viruses spread via the fecal–oral route through contaminated water, food, and poor sanitation—conditions prevalent in endemic regions such as South Asia [6]. HAV induces predominantly cytotoxic T‐cell‐mediated hepatocyte injury, while HEV triggers innate immune dysregulation; together, they may produce an amplified inflammatory response. The clinical manifestations of dual infection include jaundice, fever, fatigue, and hepatomegaly, and can progress to serious complications such as fulminant hepatic failure, with mortality exceeding that of single‐virus infection [2].

There is a growing body of case reports describing HAV and HEV co‐infection. One case from Pakistan described a 32‐year‐old male who presented with jaundice, abdominal pain, hepatomegaly, and grade 2 encephalopathy, progressing to acute liver failure; he did not survive due to unavailability of liver transplantation [11]. In another report from Lahore, Pakistan, an 8‐year‐old girl developed grade III hepatic encephalopathy secondary to dual HAV/HEV infection and, despite supportive care and broad‐spectrum antibiotics, deteriorated to grade IV encephalopathy and died within 7 days of admission [12]. An atypical presentation was documented in India, where a 32‐year‐old male developed aseptic meningitis as a complication of co‐infection [13]. In contrast, our patient did not develop significant systemic complications; her course was limited to acute‐onset jaundice, nausea, vomiting, anorexia, and deranged liver function tests.

A study of 78 children with acute viral hepatitis found that, in addition to jaundice, 82.1% of them had fever, 98.7% had hepatomegaly, and 39.7% had splenomegaly [14]. Another Egyptian study found that 92.3% of children suffering from HAV infection (mean age 7.1 years) had jaundice, 20.8% had hepatomegaly, and 2.7% had hepatosplenomegaly [15]. Conversely, our patient did not exhibit any of these typical symptoms, which deviated from the typical presentation except for the jaundice. The sequential development of jaundice on the fourth day of illness without preceding fever or systemic symptoms suggests a subtle onset, differing from more classic presentations with prodromal febrile illness.

A unique aspect of this case was the liver ultrasound finding of a “starry sky” pattern. Hyperechoic portal triads caused by inflammation, periportal edema, and decreased liver parenchymal echogenicity that highlight the portal venule walls on ultrasonography are known as “starry sky appearances” [16]. A study from the Radiology Department of King Edward Medical University, Pakistan, found that 63.8% of the starry sky appearance was seen in the pediatric population with acute HAV or HEV infection, the third most frequent radiological finding among these patients [17].

The treatment of HAV and HEV infections is nonspecific, with supportive therapy. That's why prevention remains the most important method to eliminate the burden of these infections. Vaccines are also available for both HAV and HEV. In addition, the two most crucial preventive strategies for HAV and HEV are the supply of clean drinking water and improving the standards of hygienic human waste disposal [18, 19].

## Author Contributions

**Umaimah Batool Mirza:** methodology, project administration, supervision, visualization, writing – original draft, writing – review and editing. **Habib Ul Rahman Kalhoro:** investigation, project administration, writing – original draft, writing – review and editing. **FNU Umna:** project administration, writing – original draft, writing – review and editing. **Ahmed Asad Raza:** project administration, writing – original draft, writing – review and editing. **Abedin Samadi:** project administration, writing – original draft, writing – review and editing.

## Funding

The authors have nothing to report.

## Ethics Statement

The authors have nothing to report.

## Consent

The patient provided written informed consent to participate and to allow the use of anonymized clinical data.

## Conflicts of Interest

The authors declare no conflicts of interest.

## Data Availability

The datasets generated and/or analyzed during the current study are not publicly available due to patient confidentiality but are available from the corresponding author on reasonable request.
